# Direct and indirect costs of allergic and non‐allergic rhinitis to adults in Beijing, China

**DOI:** 10.1002/clt2.12148

**Published:** 2022-04-16

**Authors:** Xian Li, Xu Xu, Jingyun Li, Yanran Huang, Chengshuo Wang, Yuan Zhang, Luo Zhang

**Affiliations:** ^1^ Department of Allergy Beijing TongRen Hospital Capital Medical University Beijing China; ^2^ Department of Otolaryngology Head and Neck Surgery Beijing TongRen Hospital Capital Medical University Beijing China; ^3^ Beijing Key Laboratory of Nasal Diseases Beijing Institute of Otolaryngology Beijing China

**Keywords:** China, chronic rhinitis, direct costs, indirect costs

## Abstract

**Background:**

Chronic rhinitis is generally classified as either allergic rhinitis (AR) or non‐allergic rhinitis (NAR). There is currently no report on the economic burden of AR and NAR in Beijing, China.

**Methodology:**

A total of 1013 valid questionnaires from 448 AR patients and 565 NAR patients living in Beijing were continuously collected for investigation of the direct (e.g., drugs, medical visits) and indirect costs (absenteeism and presenteeism) from August 2020 to April 2021.

**Results and Conclusion:**

The total cost of AR and NAR was € 195.6 patient/year and € 185.3 patient/year respectively. The total societal cost for adult AR and NAR patients in Beijing is around € 440.9 and € 671.9 million per year in terms of the standardized prevalence of the diseases. The patient's level of education, disease duration, predilection time of disease, severity of symptoms and comorbidity with other allergic disease were factors that affected the economic burden on patients with rhinitis.

To the Editor

Chronic rhinitis is defined as a symptomatic inflammation of the nasal mucosa, characterized by nasal congestion, both anterior and posterior rhinorrhea, some sneezing, and nasal itching for at least 1 h daily for a minimum of 12 weeks per year.^1^ Chronic rhinitis can be classified as allergic rhinitis (AR), when an allergen is involved as the trigger for nasal symptoms,^2^ and non‐AR (NAR), when rhinitis occurs without clinical signs of infection (discolored secretions) and without systemic signs of allergic inflammation (allergen‐specific IgE in blood and/or positive skin prick test).^1^ A longitudinal study from 18 major cities in China has shown that the standardized prevalence of adult AR and NAR in Beijing was 11.5% and 18.5%, respectively.^3^ Considering the high prevalence of AR and NAR and the impact on general well‐being, AR and NAR pose a huge economic burden due to both direct costs (e.g., drugs, medical visits) and indirect costs (absenteeism and presenteeism). However, to date, data on the economic burden of AR and NAR in patients in Beijing are not available.

In order to estimate the direct and indirect costs of AR and NAR patients in Beijing, we conducted a retrospective study in which questionnaires (provided as Supplementary information ‐ Additional file 1) filled by patients with AR and NAR referred to the Department of Allergy, Beijing Tongren Hospital from August 2020 to April 2021, were assessed. The final costs were expressed in Euros (€), based on an average exchange rate in 2020 of 7.8683 Chinese Yuan/1 €, as published by National Bureau of Statistic of China. Details on the methods and materials are provided as Supplementary information ‐ Additional file 2.

A total of 1013 valid questionnaires were obtained. The demographic and clinical characteristics of 448 AR patients and 565 NAR patients are shown in Table 1. In 2020, according to the report of the National Bureau of Statistics of the People's Republic of China (http://www.stats.gov.cn/), the average disposable income in Beijing was € 8824.4, for working 251 days, and the per capita daily salary in Beijing was € 35.2. This information allowed us to calculate the indirect cost of AR or NAR patients. The economic burden of each subgroup of AR and NAR patients is shown in Table 2. The total cost in AR and NAR patients was € 195.6 patient/year and € 185.3 patient/year, respectively. Based on the findings of Wang et al.^3^ that the standardized prevalence of adult AR and NAR in Beijing was separately 11.5% and 18.5% and there were 19.6 million adult inhabitants in Beijing according to the National Bureau of Statistics, we estimated that the total yearly cost for adult AR and NAR patients in Beijing was € 440.9 and € 671.9 million, respectively. Patients with bachelor's degrees incurred significantly higher presenteeism costs than those with upper secondary education (*P* = 0.042). The total cost (*P* = 0.000), total indirect cost (*P* = 0.046) and presenteeism cost (*P* = 0.034) to AR and NAR patients with a “persistent” disease course were higher than those with “intermittent” disease course. For patients with “perennial” disease, both direct and indirect costs were significantly higher than for patients with “seasonal” disease (*P* < 0.05). The severity of symptoms was the most important factor affecting the cost to patients; with more severe the symptom is, the higher the economic burden on patients (*P* < 0.05). Compared to patients without comorbidities, patients with self‐reported asthma, Allergic conjunctivitis (AC) and/or Atopic dermatitis (AD) had higher direct costs (*P* = 0.006) and total costs (*P* = 0.000).

**TABLE 1:** Characteristics of the participants

	AR (*N* = 448)	NAR (*N* = 565)
Course of rhinitis (years), median (IQR)	4.0 (2.0–6.0)	3.0 (2.0–5.0)
Gender, N (%)		
Male	196 (43.8%)	252 (44.6%)
Female	252 (56.3%)	313 (55.4%)
Age (years), N (%)		
18–29	137 (30.6%)	160 (28.3%)
30–44	274 (61.2%)	340 (60.2%)
45–65	37 (8.3%)	65 (11.5%)
Education, N (%)		
Lower secondary education and below	13 (3.0%)	14 (2.5%)
Upper secondary education	27 (6.0%)	22 (3.9%)
Junior college education	74 (16.4%)	92 (16.3%)
Bachelor education	199 (44.4%)	279 (49.3%)
Master education and above	135 (30.2%)	158 (28.0%)
Disease duration, N (%)		
Persistent	216 (48.2%)	263 (46.6%)
Intermittent	232 (51.8%)	302 (53.4%)
Predilection time, N (%)		
Perennial	180 (40.2%)	227 (40.1%)
Seasonal	268 (59.8%)	338 (59.9%)
Severity, N (%)		
Mild	48 (10.7%)	85 (15.0%)
Moderate/Severe	400 (89.3%)	480 (84.9%)
Treatment, N (%)		
Antihistamines	401 (89.5%)	482 (85.3%)
Intranasal corticosteroids	186 (41.5%)	232 (41.1%)
Nasal irrigation	217 (48.4%)	299 (52.9%)
Others	90 (20.1%)	95 (16.8%)
Comorbidities, N (%)		
Self‐reported asthma	45 (10.0%)	29 (5.1%)
Self‐reported allergic conjunctivitis	67 (15.0%)	48 (8.5%)
Self‐reported atopic dermatitis	38 (8.5%)	39 (6.9%)
Smoking status, N (%)		
No smoking	334 (74.5%)	496 (87.8%)
Smoking	114 (25.5%)	69 (12.2%)

Abbreviations: AR, Allergic rhinitis; NAR, Non‐allergic rhinitis.

**TABLE 2:** Subgroup analyses of patients with Chronic rhinitis (CR), median costs per individual/year in Euros (€)

	Direct cost (€), median (IQR)	Indirect cost (€), median (IQR)			Total cost (€), median (IQR)
		Absenteeism	Presenteeism	Total	
Types of rhinitis					
AR	167.7 (152.4–196.5)	175.8 (70.3–246.1)	35.2 (17.6–70.3)	63.3 (21.1–161.7)	195.6 (166.4–258.7)
ARA	190.1 (167.5–212.7)	246.1 (140.6–351.6)	35.2 (12.3–63.3)	181.1 (57.1–351.6)	225.9 (174.5–359.6)
NAR	167.7 (152.4–190.0)	158.2 (70.3–246.1)	35.2 (21.1–79.1)	70.3 (28.1–175.8)	185.3 (160.1–234.4)
*P* value^a^	0.381	0.335	0.130	0.269	0.065
Gender					
Male	167.7 (152.0–190.1)	175.8 (70.3–246.1)	35.2 (19.3–87.9)	70.3 (28.1–175.8)	190.1 (160.1–257.2)
Female	167.7 (152.4–190.1)	175.8 (105.5–246.1)	35.2 (21.1–73.2)	63.3 (21.1–167.0)	190.0 (161.3–235.0)
*P* value	0.129	0.458	0.266	0.407	0.887
Age (years)					
18–29	167.5 (152.4–185.3)	105.5 (70.3–246.1)	35.2 (17.6–70.3)	49.2 (21.1–140.6)	190.0 (166.4–255.9)
30–44	167.7 (152.4–190.3)	175.8 (79.1–325.2)	35.2 (21.1–87.9)	70.3 (28.1–175.8)	190.1 (160.1–246.8)
45–65	167.7 (152.2–190.1)	175.8 (158.2–351.6)	35.2 (14.1–52.7)	52.7 (21.1–210.9)	182.1 (152.7–219.6)
*P* value	0.328	0.094	0.240	0.253	0.119
Education					
Lower secondary education and below	167.7 (152.0–219.6)	121.2 (86.0–207.1)	29.9 (7.0–89.7)	29.9 (7.0–89.7)	169.4 (160.1–219.6)
Upper secondary education	183.4 (155.0–200.0)	158.2 (114.3–228.5)	21.1 (17.6–80.9)	80.9 (17.6–179.3)	190.1 (172.3–226.8)
Junior college education	167.7 (160.1–190.1)	246.1 (140.6–351.6)	21.1 (12.3–52.7)	35.2 (14.1–256.7)	177.7 (160.1–217.2)
Bachelor education	167.7 (153.0–195.4)	105.5 (70.3–246.1)	35.2 (17.6–73.8)^b^	70.3 (28.1–176.7)	201.3 (166.9–264.0)
Master education and above	167.7 (160.1–190.1)	175.8 (70.3–246.1)	38.7 (21.1–93.2)	59.8 (28.1–131.8)	197.0 (167.7–265.4)
*P* value	0.694	0.149	**0.042**	0.378	0.095
Disease duration					
Persistent	173.5 (152.4–195.3)	175.8 (70.3–351.6)	35.2 (21.1–87.9)	70.3 (28.1–202.2)	197.0 (167.5–260.5)
Intermittent	167.5 (152.5–190.1)	175.8 (70.3–246.1)	31.6 (17.7–73.0)	56.3 (21.1–141.5)	181.2 (159.0–230.9)
*P* value	0.075	0.372	**0.034**	**0.046**	**0.000**
Predilection time					
Perennial	173.7 (152.9–195.3)	175.8 (105.5–351.6)	54.5 (26.4–112.5)	98.4 (31.6–210.9)	196.3 (167.2–265.4)
Seasonal	167.5 (152.0–189.9)	140.6 (70.3–246.1)	28.1 (17.6–63.3)	52.7 (21.1–126.6)	184.0 (160.1–234.0)
*P* value	**0.000**	**0.028**	**0.000**	**0.001**	**0.001**
Severity					
Mild	152.9 (152.0–175.0)	52.7 (35.2–96.7)	21.1 (14.1–39.6)	28.1 (17.6–72.1)	166.8 (152.0–199.1)
Moderate/Severe	167.7 (152.4–190.1)	175.8 (87.9–246.1)	35.2 (21.1–73.8)	70.3 (28.1–175.8)	193.9 (167.3–257.5)
*P* value	**0.000**	**0.000**	**0.011**	**0.001**	**0.000**
Comorbidities					
No	167.5 (152.4–190.1)	175.8 (70.3–246.1)	35.2 (21.1–73.8)	63.3 (24.6–160.0)	186.0 (160.1–236.3)
Self‐reported asthma, AC and/or AD	168.4 (152.9–200.0)	175.8 (105.5–351.6)	35.2 (17.6–84.4)	70.3 (23.7–214.5)	202.7 (167.7–281.4)
*P* value	**0.006**	0.065	0.970	0.395	**0.000**
Smoking status					
No smoking	167.7 (152.9–190.7)	140.6 (70.3–246.1)	35.2 (21.1–73.8)	63.3 (21.1–147.7)	190.1 (165.1–240.9)
Smoking	167.7 (152.0–190.1)	246.1 (105.5–351.6)	38.7 (17.6–73.0)	70.3 (35.2–272.5)	202.1 (160.1–281.7)
*P* value	0.378	**0.022**	0.972	0.067	0.260

*Note*: *p* < 0.05 was shown in bold.

Abbreviations: ARA, Allergic rhinitis combined with asthma; AC, Allergic conjunctivitis; AD, Atopic dermatitis.

^a^ P value is the comparison between “AR” and “NAR”.

^b^ *p* < 0.05 compared with upper secondary education.

Our results showed that the patient's level of education, disease duration, predilection time of disease, severity of symptoms, and comorbidity with other self‐reported allergic disease were factors that affected the economic burden on patients with rhinitis. Patients with higher education levels often take on more complex tasks in society, requiring long‐term mental work and focused attention. Moreover, some authors have suggested that sleep disorders are common among AR patients.^4^ Thus, highly educated patients are more likely to have low work efficiency during the day due to poor sleep, resulting in higher presenteeism costs. As the symptoms last longer in “persistent” and “perennial” rhinitis patients, they lead to greater disruption to the patient's work and life. The patients also usually spend more time at the hospital and use more drugs, thereby increasing both direct and indirect costs. In addition to nasal symptoms, patients with self‐reported asthma, AC, and/or AD, also have pulmonary (e.g., chest tightness, shortness of breath or difficulty breathing and wheezing), and eye (e.g., eye itching and tearing) or skin symptoms; which require attention at respiratory, ophthalmology or dermatology departments and lead to an even greater economic burden than that of rhinitis patients without comorbidities.

In our study, the direct cost (€ 167.7) is 85.7% of total costs (€ 195.6), which is in accordance with the findings of Kim and colleagues,^5^ who demonstrated that direct costs accounted for 82% of total costs for AR patients in Korea. However, the economic burden of rhinitis patients in China appears to lag far behind that of the economic burden of rhinitis patients in several European counties.^6^, ^7^, ^8^ This suggests that there is a certain gap in the level of diagnosis and treatment of rhinitis between China and western developed countries, but with the development of economy, the corresponding medical conditions will be gradually improved.

However, there are several limitations in our study. Firstly, as this is a retrospective study, there is the potential for patients being partly biased when recalling their medication. Secondly, the inclusion of NAR in our study may have ignored some subtypes of the NAR group; for example, pregnancy rhinitis and gustatory rhinitis; which may lead to underestimation of cost burden in the NAR group. In addition, due to the shortage and high cost of allergen vaccines, the popularity of immunotherapy in China is limited. Therefore, it is difficult to completely cover patients who received immunotherapy in the past or during the current investigation. Indeed, if the patients suffering from AR choose immunotherapy, the annual economic burden will increase significantly.^9^ Thirdly, the last update of the used per capita daily salary and reference prices were based on the statistics of 2020, negating a proportion of patients visiting our hospital in the first quarter of 2021 and therefore the annual disease burden may also be underestimated.

To our knowledge, this is the first study to report the economic costs of AR and NAR in the Beijing Chinese population. Further studies are needed to investigate the costs of AR and NAR in the general Chinese population.

## CONFLICT OF INTEREST

The authors report no competing interests.

## AUTHOR CONTRIBUTIONS

Jingyun Li, Yanran Huang and Chengshuo Wang collected the data. Xian Li and Xu Xu wrote the manuscript. Yuan Zhang and Luo Zhang designed and supervised the study.

## CONSENT FOR PUBLICATION

All the authors agree on publishing the submitted document.

## SUBMISSION DECLARATION

This manuscript is original, has not been published before, is currently not being considered for publication elsewhere, and has not been posted to a preprint server.

## Supporting information

Supplementary Information 1

Supplementary Information 2
