# Supplementary material for: Direct and indirect costs of allergic and non‐allergic rhinitis to adults in Beijing, China
Source: Clin Transl Allergy. 2022 Apr 16;12(4):e12148. doi: 10.1002/clt2.12148 (PMC9012971; doi:10.1002/clt2.12148)
Supplement: Supplementary file 1 — Supplementary Information 1 [file CLT2-12-e12148-s001.docx]

**慢性鼻炎患者问卷调查表**

**姓名： 性别：男 / 女 年龄： 民族：**

**出生地： 现居住城市： (居住半年以上)**

**填写时间: 年 月 日**

**联系电话： 学历：**□初中及以下 □高中 □专科 □本科 □硕士及以上

**病史回顾：**（请您回答以下问题，并在相应选项前面的“□” 内划“√”）

1. **您的鼻部不适已持续了多长时间？** □年 / □月
2. **您的鼻炎相关症状的发作时间：**

**A、在一年内持续发作的时间：**□＜4周 □≥4周

**B、在一周内持续发作的时间：**□＜4天 □≥4天

**3. 您的鼻部不适对您日常生活的总体困扰程度如何？**

□没有困扰（0分）；□轻度困扰（1分）；□中度困扰（2分）；□严重困扰，以致不能做事（3分）

**4. 您的鼻炎发作是呈现：**

□常年性发作； □季节性发作

**5. 是否伴有其他过敏性疾病？**

□ 无 □ 哮喘 □ 过敏性皮炎 □ 蚊虫叮咬过敏

□ 过敏性结膜炎 □ 药物过敏 □ 食物过敏（如海鲜、水果、牛奶等）

**6. 您家族中是否有其他人有过敏性疾病？**

□ 无 □ 有，与您的关系是

**7. 您是否伴有其它鼻腔疾病？**

□ 无 □ 慢性鼻窦炎 □ 鼻息肉

□ 鼻腔良恶性肿瘤 □ 其它

**8. 您是否有吸烟史？**

□ 无 □吸烟，每日 支，持续 年

**9. 您是否有吸烟史？**

□ 无 □饮酒，每日 两，持续 年

**10. 您是否曾经诊断过过敏性鼻炎** □是/□否，每年因鼻炎就诊的次数____次/年

**11. 您过去一年因为鼻部不适曾经使用过以下哪些类药物(可多选):**

- - **抗组胺药**包括**口服**息斯敏___盒、氯雷他定__盒、西替利嗪__盒、盐酸非索非那定__盒、依巴斯丁__盒，其他___；和/或**鼻用**盐酸氮卓斯汀__瓶，其他___
  - **鼻用类固醇激素**包括布地奈德___瓶、丙酸氟替卡松___瓶、糠酸氟替卡松__瓶等，其他___瓶。
  - **口服激素**包括甲泼尼龙___盒、其他___盒。
  - **抗生素**如头孢类___盒、大环内酯类___盒、喹诺酮类（拜复乐）__盒、其他__等。
  - **粘液促排剂__盒**
  - **鼻腔冲洗___盒**

**12. 过去一年是否接受过过敏原检查？**

□ 无

□ 有， □ 皮肤点刺试验 次，每次平均 元；

□ 抽血过敏原检测 次，每次平均 元。

**13. 过去一年是否曾经/正在接受脱敏治疗？**

□ 无

□ 皮下脱敏治疗 月，合计约 元；

□ 舌下脱敏治疗 月，合计约 元。

**14. 过去一年您是否接受过鼻窦CT或者核磁检查？**

A: CT： □ 无 □ 有，共计 次

B: 核磁： □ 无 □ 有，共计 次

**15.过去一年您是否因鼻部不适而曾经住院治疗？**

□ 无

□ 有，住院 次，每次平均总花费 元。

**16. 过去一年您是否因鼻部疾病而手术治疗？**

□ 无

□ 有，共计手术 次，每次平均总花费 元。

**17.过去一年您是否曾经因为鼻部不适而误工/学？**

□ 无

□ 有，平均每年 天。

**18. 过去一年您是否因为鼻部不适而影响学习/工作效率及表现？**

□ 无

□ 有 天/年,

请用垂直竖线“|”在以下对应的标尺上标出影响程度的分数：

无0（完全不影响） 严重10（完全无法工作学习）

**谢谢您的认真填写！☺**

**Questionnaire for patients with chronic rhinitis**

**Name: Sex: Male/Female Age: Ethnicity:**

**Birth place: City of residence: (Lived here for more than half a year) Date: Day Month Year**

**Phone number:**

**Education:** □Lower secondary education and below □Upper secondary education

□Junior college education □Bachelor’s degree education □Master’s degree education and above

**Medical history review:**

(Please answer the following questions and mark “√” in the“□” in front of the corresponding option)

1. **How long has your nasal discomfort lasted?**

Month Year

1. **The onset time of your nose-related symptoms:**
2. Duration of onset of symptoms within one year: □ < 4 weeks □ ≥4 weeks
3. Duration of onset of symptoms within a week: □ < 4 days □ ≥4 days
4. **What is the general level of distress caused by your nasal symptoms to your daily life in the past year?**

□ NO (0 point) □ Mild distress (1 point) □Moderate distress (2 points) □Severe distress (3 points)

**4. What type of rhinitis episodes do you suffer from?**

□Perennial □Seasonal

**5. Is it accompanied by other allergic diseases?**

□ No □ Asthma □ Allergic dermatitis

□ Mosquito bite allergy □ Allergic conjunctivitis □ Drug allergy

□ Food allergy (such as seafood, fruit, milk, etc.)

**6. Does anyone else in your family have any allergic disease?**

□ No □ Yes, their relationship with you is­­­­

**7. Do you have any other nasal disease?**

□ No □ Chronic sinusitis □ Nasal polyps

□ Other tumors of the nasal cavity □ Other

1. **Do you have a history of smoking?**

□ No □Smoking, cigarettes/a day, last year

**9. Do you have a history of drinking?**

□ No □ Drinking, taels/a day, last year

**10. Have you been diagnosed with allergic rhinitis?** □YES □NO, Number of visits per year for rhinitis ____ times/year.

**11.Which of the following medicines have you used due to nasal discomfort in the past year (multiple choices available) ?**

- - **Oral/ intranasal antihistamines:** Aspismin____boxes, Loratadine (Carretan)___boxes, Cetirizine (Zitermine/Befen)___boxes, Fexofenadine hydrochloride___boxes, Ebastine (Carestine)___boxes, etc.
  - **Nasal corticosteroids:** Budesonide (Ranocotte)____bottles, Fluticasone propionate (Fushuliang)___bottles, Fluticasone furoate (Nesunar)____bottles, etc.
  - **Oral hormones:** Methylprednisolone (Eugene/Metrole)____boxes, Acetate prednisolone___boxes, etc.
  - **Antibiotics:** Cephalosporins___boxes, Macrolides (Azithromycin, Clarithromycin, Erythromycin)___boxes, Quinolones (Befole)___boxes, etc.
  - **Mucus promoting agent:** Chernob, Ouloma,___boxes, etc.
  - **Nasal irrigation ___**bottles

**12. Have you been tested for sensitization to any allergens in the past year?**

□NO □Skin prick test ___times, average cost___ yuan at a time

□Serum specific IgE for allergens __times, average cost __yuan at a time

**13. Have you received allergy immunotherapy in the past year?**

□ No

□ Yes, subcutaneous desensitization treatment ____months, total costs____yuan

□ Yes, sublingual desensitization treatment ____months, total costs___yuan

**14. Have you had a sinus CT or MRI examination in the past year?**

A. CT: □No □Yes, Total ___times

B. MRI: □No □Yes, Total ___times

**15. Have you undergone surgery for nasal discomfort in the past year?**

□ No

□ Yes, surgeries in total, average cost ___at a time

**16.** **Have you had surgery for any nasal problems in the past year?**

□No

□Yes, total number of operations ___, average cost ___at a time

**17. Have you ever missed work/school because of nasal discomfort in the past year?**

□No

□Yes, averaged ___days per year

**18. Have you suffered from nasal discomfort that affected your study/work efficiency and performance in the past year?**

□No

□Yes, ___days per year, please mark the degree of discomfort with “|” on the following line.

**None More than I can image**


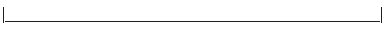


**Thank you for filling this form in carefully! ☺**
