# Supplementary material for: Direct and indirect costs of allergic and non‐allergic rhinitis to adults in Beijing, China
Source: Clin Transl Allergy. 2022 Apr 16;12(4):e12148. doi: 10.1002/clt2.12148 (PMC9012971; doi:10.1002/clt2.12148)
Supplement: Supplementary file 2 — Supplementary Information 2 [file CLT2-12-e12148-s002.docx]

**METHODS AND MATERIALS**

**Participants**

This study was approved by the ethics committee of Beijing Tongren Hospital, and all patients provided signed written informed consent prior to entry into the study. Patients who visited the Department of Allergy, Beijing Tongren Hospital from August 2020 to April 2021 were eligible to join the study if they met the following criteria: (1) age from 18 to 65 years; and (2) presence of rhinitis, which was diagnosed as AR or NAR based on the criteria of the Allergic Rhinitis and its Impact on Asthma (ARIA) consensus statement or Non-allergic rhinitis: Position paper of the European Academy of Allergy and Clinical Immunology, respectively [1, 2]. Briefly, individuals who had experienced two or more nasal symptoms (rhinorrhea, nasal congestion, nasal itching, sneezing with or without ocular symptoms for at least 1 hour daily for a minimum of 12 weeks per year and with positive SPT or serum specific IgE(sIgE) were included in the AR group; and patients suffering from rhinitis without clinical evidence of endonasal infection and without systemic signs of allergic inflammation (serum sIgE and/or positive SPT results) were included in the NAR group. The exclusion criteria for the study included i) chronic rhinosinusitis and/or nasal polyposis as defined by the European position paper on rhinosinusitis and nasal polyps [3], ii) any respiratory infection in the previous 4 weeks, and iii) CT scan showing any opacification in the nasal cavity or sinuses.

**Assessment of demographics, clinical history of rhinitis and direct and indirect costs.**

Both groups of patients with AR and NAR were asked to fill in a questionnaire to assess demographic and medical history. The questionnaire was only filled on paper, in order to reduce any bias of preference between paper and electronic versions [4]. Additionally, a researcher assisted the patient to in filling the questionnaire only when the patient couldn’t understand a question correctly, and any questionnaire that was not answered completely was regarded as invalid and not included in any subsequent analyses.

***Demographic and clinical assessment***

The questionnaire was filled in once and included questions detailing the patient’s demographic data, severity of the disease, disease duration, predilection time, asthma history, and questions related to direct costs and indirect costs. The severity of the disease was reported by the patients, and classified as being intermittent (<4 days/week or <4 weeks/year) or persistent (≥4 days/week and ≥4 weeks/year) according to the frequency of symptoms and based on the criteria of the Allergic Rhinitis and its Impact on Asthma (ARIA) consensus statement. The classification of seasonal AR (the onset of symptoms is seasonal; aeroallergens mainly including pollen) and perennial AR (the onset of symptoms is year-round; allergens including dust mites, animal dander, etc) was based on the Chinese Society of Allergy Guidelines for Diagnosis and Treatment of Allergic Rhinitis [5].

***Direct cost assessment***

Direct costs were assessed from the questionnaires, with a recall period of the past year. Reference prices were obtained from the retail prices of Beijing Tongren Hospital in 2020. Direct costs are represented by healthcare costs, such as medication use, visits to healthcare providers, hospital stay, cost of medical examinations and tests, etc. The total of all these costs was calculated as the final direct cost.

***Indirect cost assessment***

Indirect costs included the costs for absenteeism (absence at work due to illness) and presenteeism (decreased performance due to a disease). For absenteeism in the past year, the cost was calculated by multiplying days lost from working time by the average salary of an adult habitant in Beijing per day:

absenteeism= days lost from working time$\times$ $\frac{\text{averaged }\text{disposable}\text{ }\text{income}\text{ }\text{of}\text{ }\text{Beijing}\text{ }\text{in}\text{ 2020}}{\text{working}\text{ }\text{days}\text{ }\text{in}\text{ 2020}}\text{ }$

Similarly, presenteeism was calculated as:

days of lost productivity$\times\frac{\text{averaged }\text{disposable}\text{ }\text{income}\text{ }\text{of}\text{ }\text{Beijing}\text{ }\text{in}\text{ 2020}}{\text{working}\text{ }\text{days}\text{ }\text{in}\text{ 2020}}\times\frac{\text{grades of disease severity}\text{ (0-10)}}{\text{10}}$.

All costs were measured in Chinese Yuan (CNY) and then converted to Euros (€), based on an exchange rate of 7.8683 CNY = 1 € (average exchange rate in 2020 published by National Bureau of Statistics of China).

**Statistical analysis**

All statistical analyses were performed using the SPSS 26.0 software (IBM Corp.). The Kolmogorov-Smirnov test was used to evaluate the normality of the data and the non-normally distributed continuous variables were expressed as median and interquartile range. Manny-Whitney U 2-tailed test was used for between-group comparison and a Kruskal-Wallis H test was used to assess significant intergroup variability among more than 2 groups. Statistical significance was set at a *P* value＜0.05.

**Extra references:**

E1. Bousquet J, Khaltaev N, Cruz AA, Denburg J, Fokkens WJ, Togias A, et al. Allergic Rhinitis and its Impact on Asthma (ARIA) 2008 update (in collaboration with the World Health Organization, GA(2)LEN and AllerGen). Allergy. 2008;63 Suppl 86:8-160.

E2. Hellings PW, Klimek L, Cingi C, Agache I, Akdis C, Bachert C, et al. Non-allergic rhinitis: Position paper of the European Academy of Allergy and Clinical Immunology. Allergy. 2017;72(11):1657-65.

E3. Fokkens WJ, Lund VJ, Hopkins C, Hellings PW, Kern R, Reitsma S, et al. European Position Paper on Rhinosinusitis and Nasal Polyps 2020. Rhinology. 2020;58(Suppl S29):1-464.

E4. Juniper EF, Langlands JM, Juniper BA. Patients may respond differently to paper and electronic versions of the same questionnaires. Respir Med. 2009;103(6):932-4.

E5. Cheng L, Chen J, Fu Q, He S, Li H, Liu Z, et al. Chinese Society of Allergy Guidelines for Diagnosis and Treatment of Allergic Rhinitis. Allergy Asthma Immunol Res. 2018;10(4):300-53.
